# Effect of Cardiovascular Endurance Training on the Exercise Capacity and Endurance in Children With Cerebral Palsy

**DOI:** 10.7759/cureus.61595

**Published:** 2024-06-03

**Authors:** Rochelle Tauro, Sankar Ganesh, Jenniefer G Vincent

**Affiliations:** 1 Physiotherapy, St. John's Medical College, Bangalore, IND

**Keywords:** aerobic exercises, cardiovascular training, exercise capacity, endurance training, cerebral palsy

## Abstract

Background: Cerebral palsy (CP) is non-progressive brain damage that occurs before, during, or shortly after birth. CP is associated with poor physical fitness, which is linked to health problems and the development of secondary illnesses like obesity, cardiovascular disease, and diabetes. Compared to healthy peers without CP, children with CP have considerably lower VO2 peaks, which reduces their performance and aerobic capacity.

Objective: This study aimed to evaluate changes in exercise capacity and endurance among children with CP, as well as fatigue levels among their parents and caregivers, after participation in cardiovascular endurance training.

Methodology: This study included 16 children aged 7-12 years with CP (Gross Motor Function Classification System levels I, II, or III). Participants completed a 12-week cardiovascular endurance program consisting of 60-minute sessions three times weekly designed to achieve 64-95% of their heart rate maximum,based on the American College of Sports Medicine guidelines. Pre- and post-intervention measurements were recorded for the following: distance covered in a six-minute walk, maximal oxygen consumption (VO2 max) level, Early Activity Scale for Endurance rating, and Patient-Reported Outcomes Measurement Information System (PROMIS) Pediatric Fatigue Scale score and PROMIS Parent Proxy Scale and Fatigue Scale scores.

Result: Upon completing the cardiovascular endurance training, the distance covered during a six-minute walk improved by 20.95 points, resting heart rate by 5.19 points, VO2 max by 0.06 points, Early Activity Scale for Endurance by 4.06 points, PROMIS Pediatric Fatigue Scale by 7.29 points, PROMIS Parent Proxy Scale by 6.81 points, and PROMIS Fatigue Scale by 5.07 points. The maximum heart rate also showed a slight improvement of 0.33 points (p<0.01).

Conclusion: A structured exercise protocol aimed at improving cardiovascular endurance can benefit children with CP by improving their exercise capacity and endurance, which in turn can help decrease fatigue levels among their parents and caregivers.

## Introduction

Cerebral palsy (CP) is non-progressive brain damage that occurs before, during, or shortly after birth and leads to variable degrees of muscle weakness, muscle contracture, stiffness, and decreased balance and coordination. Thus, people with CP often lack the functional capacity to engage in rigorous activities such as running, leaping, climbing, and cycling [1]. These limitations can lead to poor physical fitness and the subsequent development of secondary illnesses like obesity, cardiovascular disease, and diabetes. Yet, studies on cardiorespiratory endurance training to improve the exercise capacity and endurance in children with CP are scarce. This study therefore aims to evaluate the exercise capacity and endurance in this population and how improvements in these areas can have important effects for both the patients and their caregivers.

The functional ability of children with CP is classified using the Gross Motor Function Classification System (GMFCS), where the ability to walk without assistance is classified as level I or II, the ability to walk with assistance is classified as level III, and the inability to walk is classified as level IV or V [2,3]. Although the motor deficits associated with CP have no direct effect on cardiovascular function, children with CP classified as GMFCS level I, II, or III generally have lower VO2 peak levels, compared to healthy peers [3,4]. Studies show that aerobic training can be beneficial in the rehabilitation of children with GMFCS levels I and II [5,6]. According to Nsenga et al., brief and moderate aerobic training improves the cardiorespiratory status of children with CP, raising their VO2 peak and maximum heart rate to the same levels as their age-matched, able-bodied peers [6]. Maltais et al. further found that individuals who engage in at least 30 minutes of moderate-intensity exercise (i.e., 60-75% of HR max or 50% of VO2 peak uptake) have improved cardiac output, compared to their pre-exercise levels [7]. An eight-week physical fitness intervention in children with GMFCS levels I and II resulted in a 23% increase in their cardiorespiratory endurance [7], and a three-month intervention led to a 15% increase in cardiorespiratory endurance in those with GMFCS levels II and III [8].

The Patient-Reported Outcomes Measurement Information System (PROMIS; see https://commonfund.nih.gov/promis/index) measures patient-reported outcomes across a variety of clinically relevant factors, such as pain, fatigue, physical functioning, emotional distress, and social support and participation. These factors can substantially influence quality of life, especially among patients with chronic diseases.

## Materials and methods

Participants

This study enrolled 16 children aged 7-12 years (mean age of 10 years) diagnosed with CP (GMFCS levels I-III) by a pediatrician. Eligible participants had to be able to follow and execute simple verbal commands. Children were excluded from the study if they had hip subluxation, orthopedic surgery, neurosurgery, or botulinum toxin injection within the previous six months or a severe cardiac or respiratory condition that could affect their ability to exercise. All were informed of the study purpose and procedures, and all parents provided written informed consent and assent.

Procedure

**Figure 1:**
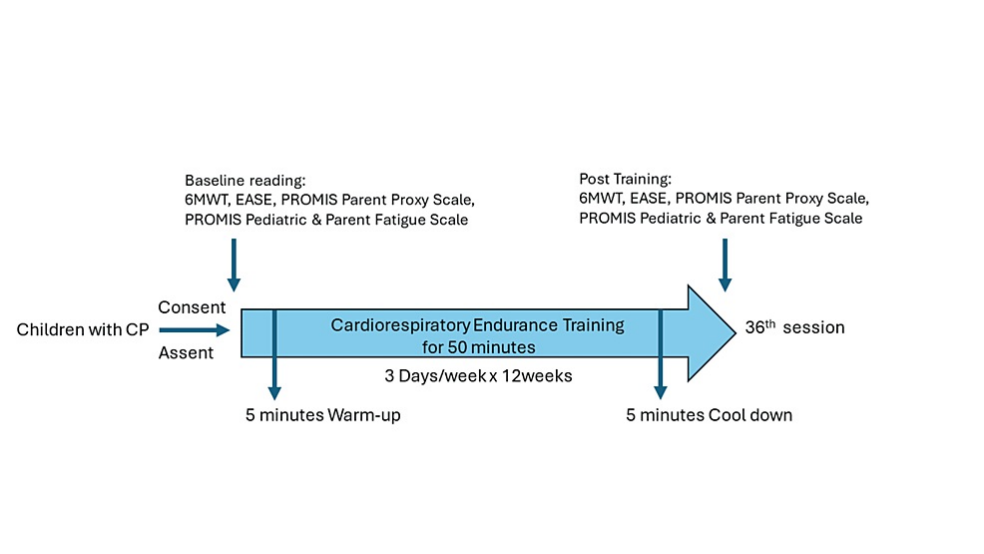
Study procedure 6MWT: six-minute walk test; EASE: Early Activity Scale for Endurance; PROMIS: Patient-Reported Outcomes Measurement Information System

This prospective study was conducted at St. John's Medical College in Bangalore, India. The study was approved by the Institutional Ethics Committee (IEC) of St. John's Medical College with IEC study reference number 283/2021. A physical therapist administered the cardiovascular training regime and collected the outcome measures. The following baseline measurements were collected: the number of meters completed in a six-minute walk test [9-11], heart rate at rest, maximum heart rate, VO2 peak, Early Activity Scale for Endurance score [12,13], and PROMIS fatigue scores for pediatric patients and their parents [14-16] (Figure 1).

Participants engaged in three training sessions per week for 12 consecutive weeks. Each session began with a five-minute warm-up that included stretching exercises for the legs, thighs, and arms, as well as breathing exercises. This was followed by 50 minutes of cardiovascular endurance training, which consisted of 20 minutes on the treadmill, 10 minutes using a gym ball, and 10 minutes of cycling or using a bicycle ergometer, ending with a five-minute cool down. The exercise intensity was aligned with the American College of Sports Medicine (ACSM) guidelines, with HRmax gradually increasing from 64% to 95% over the course of the program. Specifically, HRmax was set at 65% for the first and second weeks, 70% for the third and fourth weeks, 75% for the fifth and sixth weeks, 80% for the seventh and eighth weeks, 85% for the ninth and 10th weeks, and 90% for the 11th and 12th weeks [17]. Throughout the program, the exercises were progressively intensified by increasing the number of repetitions and the complexity of the movements.

Statistical analysis

The statistical analysis was carried out for both descriptive and inferential statistics using Stata Statistical Software: Release 16.0 (2019; StataCorp LLC, College Station, Texas, United States), with a significance level of p<0.05 considered significant. Upon verifying the homogeneity of the study population, paired t-tests were used to discern any disparities between the baseline and post-training measurements. The confidence interval was established at 95%, aligning with a p-value threshold of <0.05 to ensure statistical rigor. Furthermore, the median value for the distance covered during the six-minute walk test underwent analysis utilizing the Wilcoxon signed-rank test, providing additional insights into the children's endurance performance.

## Results

**Figure 2:**
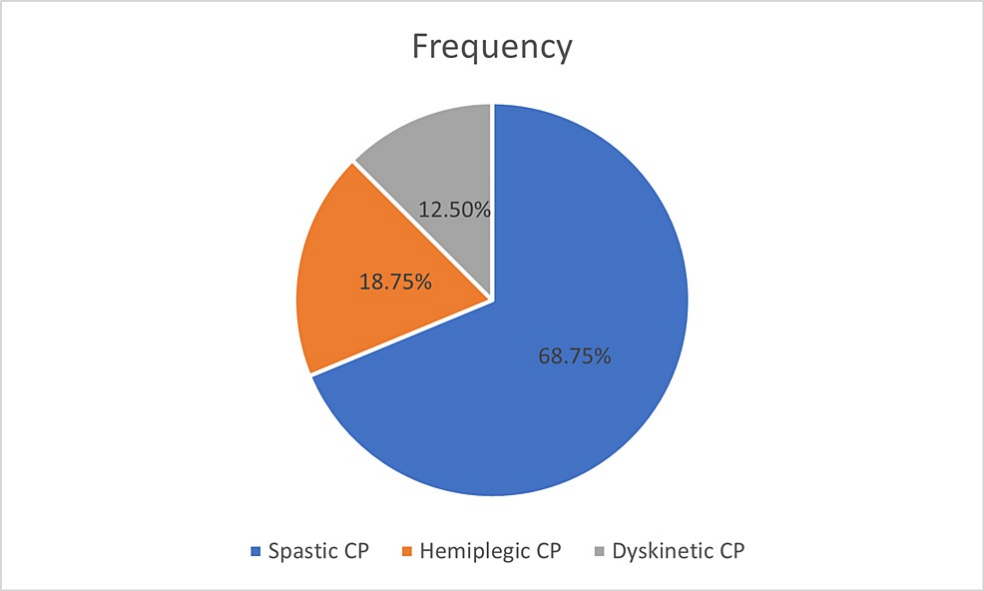
Distribution of CP children based on diagnosis The chart shows that 68.75% of the CP children were spastic CP, 18.75% were hemiplegic CP, and 12.50% were dyskinetic CP. CP: cerebral palsy

Of the 16 children (five males and 11 females), the most common diagnosis was spastic CP, followed by hemiplegic CP and dyskinetic CP (see Figure 2).

Table 1 shows that the median value of p50 for the distance covered during the six-minute walk test measured in meters increased from 49.55 m (37.55, 207) and 70.5 m (46.25, 231) showing significant changes (p<0.001).

**Table 1:** Pre- and post-test difference in six-minute walk test

Variable	N	p50	p25	p75
Pre-test	16	49.55	37.55	207
Post-test	16	70.5	46.25	231

Following cardiovascular endurance training, various improvements were observed. These include a decrease in resting heart rate by 5.2 points, an increase in VO2 peak by 0.6 points, a rise in the Early Activity Scale for Endurance by 4.06 points, as well as improvements in the PROMIS Pediatric Fatigue Scale by 6.88 points, the PROMIS Parent Proxy Scale by 6.81 points, and the PROMIS Fatigue Scale by 4.06 points. 

Table 2 shows the pre- and post-mean and standard deviations of resting and maximum heart rate values, VO2 peaks, Early Activity Scale for Endurance scores, and PROMIS scores. Results of the paired t-tests show significant improvements in resting heart rate and VO2 peak after the intervention (p<0.05). Conversely, the results for the maximum heart rate were not significant (p>0.05). As the maximum heart rate was recorded during the six-minute walking test, the participants' exercise responses differed from the pre-test measures and thus could explain the non-significance in this measure.

**Table 2:** Pre- and post-cardiorespiratory endurance training results (n=16) Analyzed using paired t-test *: statistically significant at p<0.05; PROMIS: Patient-Reported Outcomes Measurement Information System

Outcome measures	Pre-intervention mean(SD)	Post-intervention mean(SD)	Pre-post-intervention mean (95% CI)	t-value	p-value*
HR rest	103.13±7.88	97.94±5.89	5.2 (2.76,7.61)	4.6	0.000
HR max	115.75±3.47	114.25±3.80	1.5 (-0.6,3.6)	1.5	0.148
VO2 peak	17.36±1.68	17.91±1.41	-0.6 (-0.88,-0.23)	-3.6	0.003
Early Activity Scale for Endurance	14.19±1.42	10.13±1.96	4.06 (3.08,5.04)	8.8	0.000
PROMIS Pediatric Fatigue Scale	33.75±7.72	26.88±6.62	6.88 (5.96,7.79)	16.1	0.000
PROMIS Parent Proxy Scale	32.56±5.09	25.75±4.92	6.81 (5.96,7.67)	17.02	0.000
PROMIS Fatigue Scale	20±2.03	15.94±1.84	4.06 (3.61,4.52)	19.03	0.000

Both pre- and post-intervention values of the Early Activity Scale for Endurance, PROMIS Pediatric Fatigue Scale, and PROMIS Parent Proxy Scale demonstrated significant improvements (p<0.05). This suggests that physical activity interventions aimed at enhancing endurance and exercise capacity are beneficial for children with CP. Additionally, Table 2 displays a statistically significant correlation (p<0.05) between pre- and post-intervention values for the PROMIS Fatigue Scale, indicating that parents observed reduced fatigue levels as their children became more capable of independently completing tasks.

## Discussion

This study aimed to assess the impact of cardiovascular endurance training on the exercise capacity and endurance in children with CP, along with evaluating parental perceptions of their child's fatigue levels. Unlike previous research primarily focused on typically developing individuals, this study addresses the unique physical characteristics and capabilities of children with CP, filling a critical gap in the literature [8]. However, this study specifically examines the response of children with CP to cardiovascular endurance training and its impact on their fatigue levels.

A study by Maltais et al. emphasized the benefits of exercise training conducted at least 2-3 times per week for a minimum of 30 minutes at a moderate intensity, resulting in significant improvements in cardiovascular parameters [7]. In our study, we followed a similar approach to cardiovascular endurance training and observed notable outcomes among children with CP. Specifically, we found a significant reduction in resting heart rate following the intervention, indicating an improvement in cardiovascular health. The mean difference of 5.2, with a confidence interval of 2.76-7.61, demonstrated statistical significance, as indicated by the t-value of 4.6 and p-value of less than 0.001.

Although there was a slight decrease in maximum heart rate post-intervention, this change was not statistically significant. This lack of significance could be attributed to the fact that children with CP were already approaching their maximum heart rate before the intervention, as evidenced by the mean difference of 1.5 and the confidence interval of -0.6 to -3.6, with a t-value of 1.5 and a p-value of 0.148. However, a significant improvement in peak oxygen consumption was observed post-intervention, indicating enhanced aerobic capacity among the children. The mean difference of -0.6, with a confidence interval of -0.88 to -0.23, yielded a t-value of -3.6 and a p-value of 0.003, confirming statistical significance. This finding aligns with previous research conducted by Nsenga Leunkeu et al., who also demonstrated significant enhancements in peak oxygen consumption, heart rate, and ventilation in children with CP who engaged in aerobic training [6].

Following the intervention, there was a notable improvement in the Early Activity Scale for Endurance, indicating a significant enhancement in the children's endurance levels. This was evidenced by a mean difference of 4.06, with a confidence interval of 3.08-5.04, yielding a high t-value of 8.8 and a p-value of less than 0.001, signifying statistical significance. Many children exhibited increased physical activity levels, greater energy levels, and a reduction in fatigue. These improvements in endurance and energy levels not only reflect positively on the children's physical well-being but also suggest an improvement in their overall quality of life.

Consistent with these objective improvements, there was also a significant decrease in scores on the PROMIS Parent Proxy Scale post-intervention. The mean difference of 6.81, with a confidence interval of 5.96-7.67, resulted in a substantial t-value of 17.02 and a p-value of less than 0.001, indicating statistical significance. This decrease in scores suggests a considerable improvement in parental perceptions of their child's fatigue levels. These perceptible changes in the child's behavior and attitude, as observed by the parents, are indicative of the tangible benefits derived from the intervention.

Moreover, there was a significant reduction in self-reported fatigue levels among the children, as reflected in decreased scores on the PROMIS Fatigue Scale post-intervention. The mean difference of 4.06, with a confidence interval of 3.61-4.52, yielded an impressive t-value of 19.03 and a p-value of less than 0.001, underscoring statistical significance. This reduction in fatigue levels indicates improved engagement and participation among the children in various activities, which in turn contributes positively to their overall well-being and quality of life.

The intervention also led to a substantial enhancement in the Early Activity Scale for Endurance, with a mean difference of 4.06 (95% CI: 3.08-5.04), showing statistical significance (t-value: 8.8, p<0.001). Many children exhibited improved physical activity levels, higher energy, and reduced fatigue [13]. Consistent with improvements in objective measures, there was a significant decrease in scores on the PROMIS Parent Proxy Scale post-intervention, indicating a mean difference of 6.81 (95% CI: 5.96-7.67) and statistical significance (t-value: 17.02, p<0.001). This suggests a considerable improvement in parental perceptions of their child's fatigue levels, reflective of observable changes in behavior and attitude [14]. Furthermore, there was a significant reduction in self-reported fatigue levels among the children, reflected in decreased scores on the PROMIS Fatigue Scale post-intervention (mean difference: 4.06, 95% CI: 3.61-4.52), with statistical significance (t-value: 19.03, p<0.001). This signifies improved engagement and participation among the children, contributing to their overall well-being [15].

The findings of the current study highlight the significant positive impact of cardiovascular endurance training on a range of physiological and subjective outcomes related to endurance and fatigue in children with CP. These results emphasize the effectiveness of tailored exercise interventions in enhancing the overall well-being and functional capacity of this demographic. The observed enhancements in various physiological measures, such as resting heart rate, peak oxygen consumption, and endurance levels, suggest that cardiovascular endurance training can lead to substantial improvements in cardiovascular health and physical endurance among children with CP.

Additionally, the study's inclusion of a diverse population spanning different levels of motor impairment, as indicated by GMFCS levels I-III, underscores the relevance and applicability of the findings across a broad spectrum of individuals with CP. This suggests that personalized interventions tailored to the specific needs and abilities of each child can yield meaningful improvements in their well-being and functional capacity. However, despite the promising results, it's important to acknowledge certain limitations of the study. The single-center design limits the generalizability of the findings to a broader population, as factors such as geographical location and institutional practices may influence the results. Additionally, the absence of a control group makes it challenging to attribute the observed improvements solely to the cardiovascular endurance training intervention, as other factors may have contributed to the outcomes. Furthermore, the small sample size may limit the statistical power of the study and increase the risk of chance findings. Therefore, caution should be exercised when interpreting the results, and further research with larger sample sizes and rigorous study designs, including control groups, is needed to validate the findings and explore the long-term sustainability of the observed benefits.

## Conclusions

Our study demonstrates the efficacy of cardiovascular endurance training in improving the exercise capacity, endurance, and fatigue levels among children with CP. Through pre- and post-intervention assessments, we observed significant improvements in key physiological parameters and subjective measures. Specifically, cardiovascular endurance training led to a notable decrease in resting heart rate, indicative of improved cardiovascular efficiency. We observed a significant enhancement in peak oxygen consumption, highlighting improved aerobic capacity among the children. Additionally, self-reported fatigue levels among the children decreased significantly, indicating enhanced engagement and participation. These findings highlight the potential of cardiovascular endurance training to enhance their overall well-being and functional capacity. However, further research could involve assessing the long-term sustainability of these improvements and optimizing exercise recommendations for this population.
